# Fungal Strains with Identical Genomes Were Found at a Distance of 2000 Kilometers after 40 Years

**DOI:** 10.3390/jof8111212

**Published:** 2022-11-16

**Authors:** Qili Zhu, Yang Lin, Xueliang Lyu, Zheng Qu, Ziyang Lu, Yanping Fu, Jiasen Cheng, Jiatao Xie, Tao Chen, Bo Li, Hui Cheng, Weidong Chen, Daohong Jiang

**Affiliations:** 1 State Key Laboratory of Agricultural Microbiology, Huazhong Agricultural University, Wuhan 430070, China; 2 Provincial Key Laboratory of Plant Pathology of Hubei Province, College of Plant Science and Technology, Huazhong Agricultural University, Wuhan 430070, China; 3 Xinyang Academy of Agricultural Sciences, Xinyang 464000, China; 4 United States Department of Agriculture, Agricultural Research Service, Washington State University, Pullman, WA 99164, USA; 5 Shenzhen Institute of Nutrition and Health, Huazhong Agricultural University, Wuhan 430070, China; 6 Shenzhen Branch, Guangdong Laboratory for Lingnan Modern Agriculture, Genome Analysis Laboratory of the Ministry of Agriculture, Agricultural Genomics Institute at Shenzhen, Chinese Academy of Agricultural Sciences, Shenzhen 518000, China

**Keywords:** *de novo* hybrid assembly, genetic diversity, genome stability, fungi, *Sclerotinia sclerotiorum*, single nucleotide polymorphisms (SNP)

## Abstract

Heredity and variation are inherent characteristics of species and are mainly reflected in the stability and variation of the genome; the former is relative, while the latter is continuous. However, whether life has both stable genomes and extremely diverse genomes at the same time is unknown. In this study, we isolated *Sclerotinia sclerotiorum* strains from sclerotium samples in Quincy, Washington State, USA, and found that four single-sclerotium-isolation strains (PB4, PB273, PB615, and PB623) had almost identical genomes to the reference strain 1980 isolated in the west of Nebraska 40 years ago. The genome of strain PB4 sequenced by the next-generation sequencing (NGS) and Pacific Biosciences (PacBio) sequencing carried only 135 single nucleotide polymorphisms (SNPs) and 18 structural variations (SVs) compared with the genome of strain 1980 and 48 SNPs were distributed on Contig_20. Based on data generated by NGS, three other strains, PB273, PB615, and PB623, had 256, 275, and 262 SNPs, respectively, against strain 1980, which were much less than in strain PB4 (532 SNPs) and none of them occurred on Contig_20, suggesting much closer genomes to strain 1980 than to strain PB4. All other strains from America and China are rich in SNPs with a range of 34,391–77,618 when compared with strain 1980. We also found that there were 39–79 SNPs between strain PB4 and its sexual offspring, 53.1% of which also occurred on Contig_20. Our discoveries show that there are two types of genomes in *S. sclerotiorum*, one is very stable and the other tends to change constantly. Investigating the mechanism of such genome stability will enhance our understanding of heredity and variation.

## 1. Introduction

Heredity and evolution are the most concerning issues of mankind. Every living individual inherits the genome of its parents, that is, a set of genetic instructions. Except for non-cellular organisms, such as RNA viruses, life has a DNA genome. Because of different genomes, species show unique biological characteristics, and all species form a colorful life world. Each species has a relatively stable genome [1,2], in the process of reproduction, parents pass the genome faithfully to their offspring so that the biological characteristics are transmitted from generation to generation. However, errors occur in the process of replication and injury repair, leading to genome variation [3,4,5,6,7]. Haploids were more susceptible to single nucleotide mutations (SNM) and mitochondrial mutations, whereas large changes to genome structure were more common in diploids [8]. On average, ~1% of an individual’s genome will be different from the human reference sequence [9] and the genome of other individuals of life is also diverse [10,11,12,13,14,15,16,17].

Heredity and variation are the core characteristics of life. The modern evolutionary theory believes that heredity is relative while variation is continuous and never stops. It is generally believed that variation is neutral, but evolution is directed by environmental factors and even affected by non-DNA sequences [18,19,20]. Variation is a double-edged sword in that excessive or rapid variation makes species lose their nature. Mutation and epimutation are the basic phenomena of life. Without variation, they cannot adapt to changing environments. Favorable mutation and epimutation play important roles in species adaptation [21,22,23,24]. Genomic variants are commonly subdivided into single nucleotide polymorphisms (SNP), small insertion and small deletion (InDel), or large-scale structural variations (SV). Due to the high frequency and the relatively dense and uniform distribution in the genome, SNP is considered to be the major variant type within the species [25]. The frequency of SNP in eukaryotic genomes depends on such characteristics as domestication and breeding history, mating system, mutation, repeat density, and recombination frequency [26,27]. Currently, the next-generation sequencing (NGS) technology is usually used to detect SNPs and InDels in genomes [28,29,30].

Fungi are eukaryotes whose cell walls are mainly composed of chitin and glucan. Usually, their vegetative bodies are branched tubular filaments and their chromosomes are haploid, and most fungi can reproduce both sexually and asexually. Fungi have become an important model organism for studying eukaryote genetics, variation, and evolution due to their small genome size, short life cycle, ease of cultivation, and ease of genetic manipulation [16,31,32,33,34,35]. Since each vegetative cell of fungi has the potential to develop independently into an individual, the fungal genome is prone to mutating and accumulating variation, and some fungi accumulate a large number of variations during vegetative growth [36,37]. Studies have also found that species in fungi with very large and long-lived individuals appear to maintain highly stable genomes within their mycelia [38,39].

*Sclerotinia sclerotiorum* is a notorious phytopathogen for its broad host range [40,41,42]. More than 700 plant species are susceptible to this fungus including rapeseed, soybean, peanuts, and tomato [43]. *Sclerotinia sclerotiorum* is typical necrotrophic and leads to significant yield loss and economic damage on many economically important crops worldwide each year. It overseasons in the soil as sclerotia, the dormant body. *Sclerotinia. sclerotiorum* could not produce any asexual spores and use ascospores to initiate its infection on plant petals and senescent or wounded tissues [43,44]. Sclerotia can also germinate myceliogenically to infect near-ground leaves, roots, and stem basal [42]. *Sclerotinia sclerotiorum* is homothallic [40] and has a powerful complicated non-self-recognition system. These characteristics drive it to undergo a clonal life cycle and limit the exchange of DNA with other populations [45,46,47], suggesting that it may lead to a divergent evolution [48]. The genome sequence of *S. sclerotiorum* strain 1980 UF-70, a single-ascospore-isolation offspring of strain 1980 [49], was first released in 2011 [40], then the original strain 1980 was re-sequenced by using Pacific Biosciences (PacBio) technology in 2017 [50], and recently the genome of strain WH6 isolated from China was also released [51]. It is not surprising that there are exaggerated differences between the genomes of these strains. 

We collected sclerotia of *S. sclerotiorum* from pinto bean seeds at Central Bean Company, Quincy, Washington (WA) in 2018 to isolate and screen beneficial mycoviruses and mycovirus-mediated hypovirulent strains. Meanwhile, we found that some single-sclerotium-isolation strains, such as strain PB4, had very similar biological phenotypes to strain 1980 which was isolated by Dr. Jim Steadman from bean culls in western Nebraska in 1980 [46]. Thus, we sequenced the genome of strain PB4 by using combined NGS and SMRT (PacBio Sequel platform) sequencing technology and surprisingly found that the genome sequence of the strain PB4 was almost identical to that of strain 1980, although the two strains were isolated for near 40 years apart and 2000 km away from each other. We further compared the genome of strains isolated from a single rapeseed field at Xinyang city, China, strains from sclerotia in Quincy, WA, USA, and 31 ascospore offspring of strain PB4, and found that three more strains from the USA were much closer to strain 1980 than to strain PB4, while Chinese strains were very diverse. The extreme variation and super-stability of the genome are presented in the same species, *S. sclerotiorum,* reminding us that there may be unknown mechanisms to balance the inheritance and variation in one specific species.

## 2. Materials and Methods

### 2.1. Isolates and Culture Methods of S. sclerotiorum

The wild-type (WT) PB strains were isolated from sclerotia mixed in seeds of pinto beans at Central Bean Company, Quincy, WA in 2018. The XY series of WT strains were isolated from diseased rapes (*Brassicae napus*) in a single field in Xinyang, Henan Province, China in 2020. Strain Ep-1PN was isolated from a diseased eggplant in Jiamusi, Heilongjiang Province, China in 1992, and strain Ep-1PNA367 is an ascospore progeny of strain Ep-1PN which was isolated in 1997 [52]. Strain DT-8 was isolated from diseased rapeseed in Yiyang, Hunan Province, China in 2007 [53]. Strain AH98 was isolated in Hefei, Anhui Province, China in 2009 [54]. Strain 1980 or Ss-1 was originally isolated from bean culls in western Nebraska by Dr. Jim Steadman, the USA in 1980 [49]. All strains were cultured on potato dextrose agar (PDA) medium at 20 °C and stored at 4 °C (Table 1).

### 2.2. Sexual Reproduction of Strain PB4

The strain PB4 was incubated in a carrot culture medium under a dark condition of 20 °C for one month and the mature sclerotia were harvested. The sclerotia were vernalized at 4 °C under dark conditions for 14 days and buried in the vermiculite to induce sexual reproduction at 20 °C. Strains PB4-01 to PB4-31 are single-ascospore-isolation offspring derived from strain PB4.

### 2.3. Extraction of Genomic DNA

The mycelia of all strains were collected 30 h post inoculation (hpi) on PDA plates with a membrane. The genomic DNA was extracted according to the previous protocol [55] and the DNA samples were dissolved in 200 µL of TE buffer (10 mM Tris, 1.0 mM EDTA, pH 8.0) before library construction.

### 2.4. Library Construction and Data Filtering

The DNA concentrations and purity were tested by a NanoDrop™ 2000 Spectrophotometer (Thermo Fisher Scientific, Waltham, MA, USA). The DNA integrity was determined by electrophoresis analysis in 1% agarose gel. The average insert size of the library for NGS (BGISEQ-500, Beijing Institute of Genomics, Wuhan, China or Illumina NovaSeq 6000, Annuoyouda Biotechnology Co., Ltd., Zhejiang, China) is 200–400 bp. The reads with length > 2000 bp were maintained for SMRT (Beijing Institute of Genomics, Wuhan, China). For quality control, the raw data of NGS were treated as follows: (1) removing the reads with a certain proportion (40% as default) of low quality (≤20) bases; (2) removing a certain proportion (40% as default) of Ns (unknown or unspecified nucleotides) and (3) removing adapter contamination and duplication contamination. The raw data produced by SMRT was treated: (1) the PacBio subreads were filtered from polymerase reads and (2) adapters and sequences less than 2000 bp were removed before *de novo* assembly.

### 2.5. Genome Assembly and Assessment

The genome draft of strain PB4 was generated by a hybrid and hierarchical assembly strategy using all clean reads from both NGS and SMRT. Flye v.2.8.3 [56] was used to complete the *de novo* genome assembly of strain PB4 with default settings. To estimate the assembly quality, high-quality reads were aligned onto the genome draft using BWA v.0.7.17 [57] with default parameters. Sambamba v.0.8.0 [58] was used for removing PCR duplicates. The low-quality reads were filtered by SAMtools v.1.11 [59] by default. The *de novo* assembly draft was polished by Pilon v.1.23 [60] with reads produced by NGS to improve the sequence accuracy. BUSCO v.5.1.2 (http://gitlab.com/ezlab/busco/, accessed on 3 September 2020) was used to evaluate the completeness of the *de novo* genome assembly with default parameters [61].

### 2.6. Gene Prediction and Annotation

The PB4 genome was annotated by MAKER v.2.30 [62] according to its annotation pipeline. Gene calls were generated using AUGUSTUS v.2.7 [63] and GeneMark v.4.33 [64]. The GO functional annotation was performed by Blast2GO (http://www.blast2go.com/b2ghome/, accessed on 3 November 2020). Homology of the pathogenicity, virulence, and effector-related proteins, cytochrome P450 proteins, and CAZymes were identified by BLASTP against the PHI database v.3.6 (http://www.phi-base.org/, accessed on 3 November 2020), CYP database (http://p450.riceblast.snu.ac.kr/intro.php, accessed on 3 November 2020) and CAZY database (http://www.cazy.org/, accessed on 3 November 2020), respectively. The effector-like proteins were identified by the following steps: (1) signal peptides (SP) were identified by SignalP v.5.0 (http://www.cbs.dtu.dk/services/SignalP/, accessed on 7 November 2020); (2) proteins containing putative glycophosphatidylinositol membrane-anchoring domains or transmembrane domains were identified by GPIsom (http://gpi.unibe.ch/, accessed on 9 November 2020) and TMHMM v.2.0 (http://www.cbs.dtu.dk/services/TMHMM/, accessed on 9 November 2020), respectively and (3) EffectorP v.1.0 (http://effectorp.csiro.au/, accessed on 9 November 2020) was used to identify potential effectors from the proteins with SP but without glycophosphatidylinositol membrane-anchoring domains and transmembrane domains. The genome of *S. sclerotiorum* strain PB4 was visualized by Circa (https://omgenomics.com/circa/, accessed on 3 March 2021).

### 2.7. Comparative Genomics

The genomic collinearity between strains PB4 and 1980 (GCA_001857865.1) was analyzed by MCscanX [65]. Snippy v.2.6 (https://github.com/tseemann/snippy/, accessed on 24 March 2021) was used to identify core SNPs and InDels between *de novo* assembled genome of PB4 and 1980 with default parameters. Assemblytics (http://assemblytics.com/, accessed on 27 March 2021) was used to analyze the alignments obtained by MUMmer’s nucmer program [66] to identify high-confidence SV, the minimum variant size is set to 50 bp and the maximum variant size is 100,000 bp. The GATK v.3.7 [67] was used to identify SNP for NGS data of all strains with the filter conditions as follows: QD < 4.0 || FS > 60.0 || MQ < 40.0 || SOR > 3.0 || MQRankSum < −12.5 || ReadPosRankSum < −8.0. These variants were annotated by snpEff v.5.1 [68].

### 2.8. Phylogenetic Analyses

A genome-wide SNP-based phylogenetic tree was constructed for all 34 strains. All sites with SNP were used for the phylogenetic analysis. MEGA v.6 [53] was used to construct a neighbor-joining phylogenetic tree with 1000 bootstraps.

## 3. Results

### 3.1. De Novo Assembly and Functional Annotations of the Genome of the S. sclerotiorum Strain PB4

**Figure 1:**
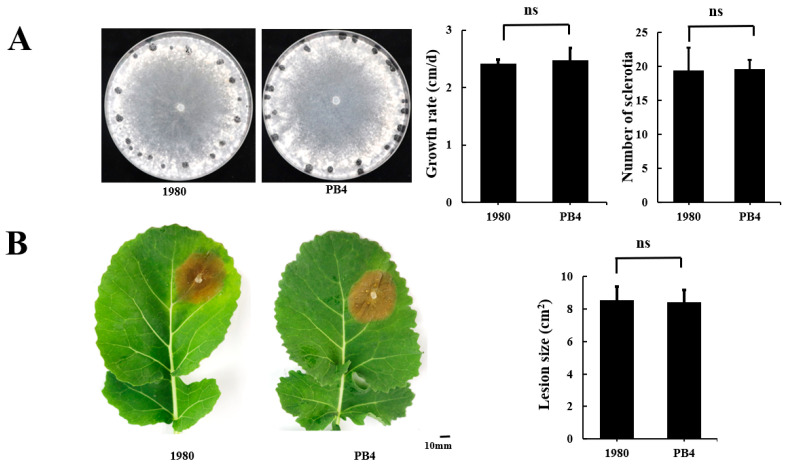
The biological characteristics of *Sclerotinia sclerotiorum* strains PB4 and 1980. (**A**) Colony morphology, hyphal growth rate, and the number of sclerotia formed. All strains were cultured on PDA plates at 20 °C for 15 days before photography. (**B**) Pathogenicity on detached leaves (*Brassicae napus*) 48 h post inoculation. ns means no significant.

**Figure 2:**
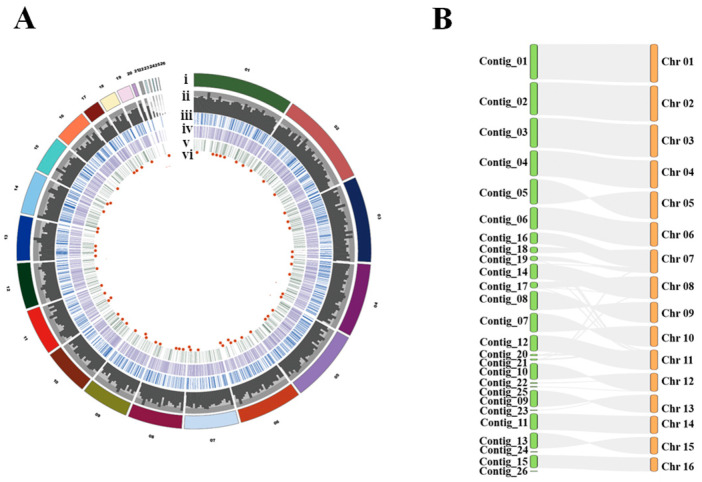
The hybrid genome assembly of *S. sclerotiorum* strain PB4 and the genomic synteny visualization of *S. sclerotiorum* strains PB4 and 1980. (**A**) Functional annotations of 26 contigs of PB4 (i), the distributions of gene density (ii), PHI-related genes (iii), cytochrome P450 (CYP) encoding genes (iv), carbohydrate-active enzymes (CAZyme) encoding genes (v), and effector encoding genes (vi) in the contigs. (**B**) The synteny of the genomes. Green colors represent the contigs of strain PB4 and orange colors represent the chromosome of strain 1980.

We isolated a strain PB4 with an extremely similar phenotype to that of strain 1980 (Figure 1A,B) and wondered how similar genomes they have. Therefore, we sequenced the genome of strain PB4 using both NGS and SMRT technologies. The sequencing reads from SMRT were used for direct assembly and those from NGS were used to correct the assembled genome. The parameters for genome assembly and polish were listed in Tables S1 and S2. The draft genome of strain PB4 consists of 26 contigs with N50 length of 2.18 Mb, a guanine-cytosine (GC) content of 41.52% and a total assembly size of 39.09 Mb (Figure 2A). The longest contig is up to 3.95 Mb. Our results showed that the genome has a 148-fold coverage by mapping the PacBio reads (Table S3). BUSCO analysis showed that 97.8% of the predicted genes were with a complete and single copy, and the proportions of fragmented and missing genes were only 0.3% and 1.7%, respectively. GeneMark and Augustus were combined to train the genome sequences and a total of 11,248 genes, 32,363 exons, and 32,358 CDS were identified by Maker using both the GeneMark and Augustus hidden Markov (HMM) model. Based on the gene annotation, 476 predicted carbohydrate-active enzymes (CAZymes) (Figure S1A), 2924 PHI-related proteins (Figure S1B), 63 *S. sclerotiorum* putative effectors (SsPEs) genes and 102 cytochrome P450 (CYP) genes were identified in the strain PB4 genome using our genome annotation pipeline (Figure 2A, details see Methods). A total of 6015 genes were classified into diverse GO functional categories (Figure S1C). Compared with the genome of strain 1980, there were seven new predicted proteins in the genome of strain PB4, three of which were predicted to have conserved domains (Figure S2).

### 3.2. The Genome Sequences of Strains PB4 and 1980 Are Almost Identical 

There are two sets of genome sequences of strain 1980, the one released in 2011 (GCA_000146945.2, version 1) was generated from strain 1980 UF-70, a single-ascospore-isolation offspring of strain 1980, by using Sanger technology, while the one released in 2017 (GCA_001857865.1, version 2) was directly generated from strain 1980 by using PacBio technology. Version 2 was used here as a reference.

The genomic synteny of strains PB4 and 1980 was quite good (Figure 2B), indicating that our assembly almost reconstituted the near chromosome-level genome of strain 1980. To our surprise, there are only 135 SNPs between strains PB4 and 1980 (Table S4), unevenly distributed on 20 contigs (ref genome of strain PB4) or 16 chromosomes (ref genome of strain 1980), and there were 48 SNPs on Contig_20 (138,000 nucleotides (nt) in length) or 54 SNPs on Chromosome 11 (2,052,000 nt in length). Other SNPs are distributed on the other 15 chromosomes of strain 1980 or other 19 contigs of strain PB4 (Figure S3A,B). A total of 572 InDels were also detected between these two genomes (215 insertions, 340 deletions and 17 compounds). A total of 40 InDels were randomly selected for verification by PCR amplification and 82.5% of them proved to be false. Considering that these two strains were isolated 40 years apart and 2000 km from each other, this phenomenon is rather rare. 

### 3.3. Distinct Population Evolution Patterns of Strains in the USA and China

Regardless of the different false positive rates caused by sequencing reads coverage, data quality, filter criteria, and so on. SNPs (532) identified using the data of NGS were 3.94-fold of those (135) identified by the combination of SMRT and NGS between strain PB4 and 1980. The MUMmer confirmed that there were 18 SVs (11 insertions and 7 deletions) between the two strains, a total of 39,420 bp length in the genome was found to be involved in (Table 2).

**Figure 3:**
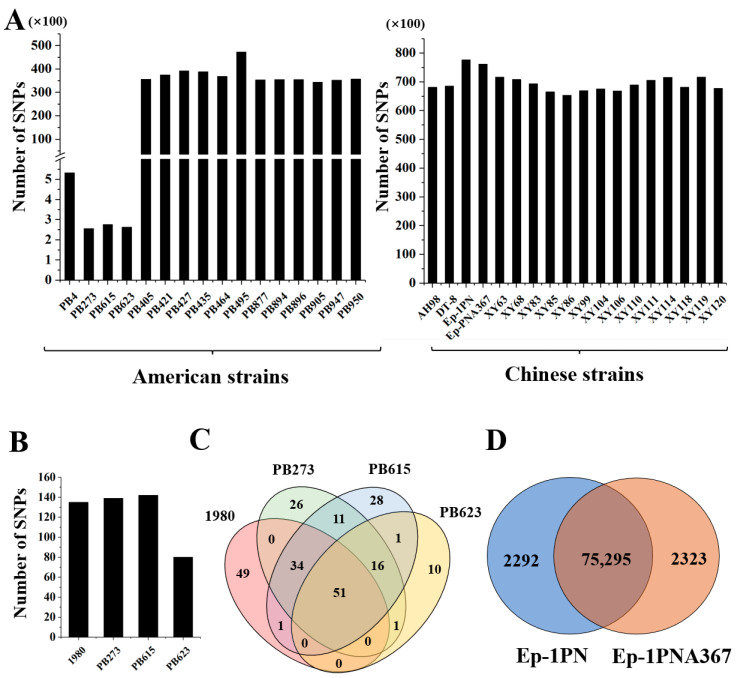
The genomic variation analysis of all *S. sclerotiorum* strains in this study. (**A**) The number of single nucleotide polymorphism (SNP) in all American and Chinese *S. sclerotiorum* strains compared with the genome of strain 1980. (**B**,**C**) The number of SNP in strains 1980, PB273, PB615 and PB623 compared with the genome of strain PB4. (**D**) The number of SNP in strains Ep-1PN and Ep-1PNA367 compared with strain 1980.

To further investigate the variation among the genomes, 14 more strains isolated from sclerotia mixed in bean seeds of America were sequenced by using NGS, and SNPs were identified by comparing with the genome of strain 1980. We found that three strains, PB273, PB615, and PB623, had even fewer SNPs than strain PB4 (532 SNPs), by 256, 275, and 262, respectively (Figure 3A). These three strains were much more similar to strain 1980 than strain PB4. The number of SNPs of the other eleven PB strains was 34,391–47,264 compared with the genome of strain 1980, and strain PB495 had the most SNPs. Thus, there exists a population that shares a similar genome with strain 1980 (Figure 3A).

Compared with the genome of strain PB4, there were 139, 142, and 80 SNPs in strains PB273, PB615, and PB623, respectively (Figure 3B), among which 67 SNPs were common in the three strains. A total of 51 SNPs were shared in strains PB273, PB615, PB623, and 1980, 48 of which were distributed on Contig_20 (Figure 3C and Figure S3A). There was no difference on Contig_20 among the four strains. Therefore, strain PB4 possesses a high frequency of SNP on Contig_20 compared with the four strains.

To further explore whether this phenomenon is universal for *S. sclerotiorum* strains at different locations, we compared the data generated by NGS of 18 Chinese strains (14 from a single rapeseed field at Xinyang city and 4 from different locations) with strain 1980 (Figure 3A) and found that there were much more SNPs (65,317–77,618) in all Chinese strains than in the PB strains (Figure 3A). Strain XY86 had the fewest SNPs, while strain Ep-1PN and its sexual offspring Ep-1PNA367 had the most SNPs among all strains tested. Strains Ep-1PN and Ep-1PNA367 shared 75,295 SNPs with 2292 and 2323 unique, respectively (Figure 3D), suggesting the genome represented by strain Ep-1PN changed very fast. 

**Figure 4:**
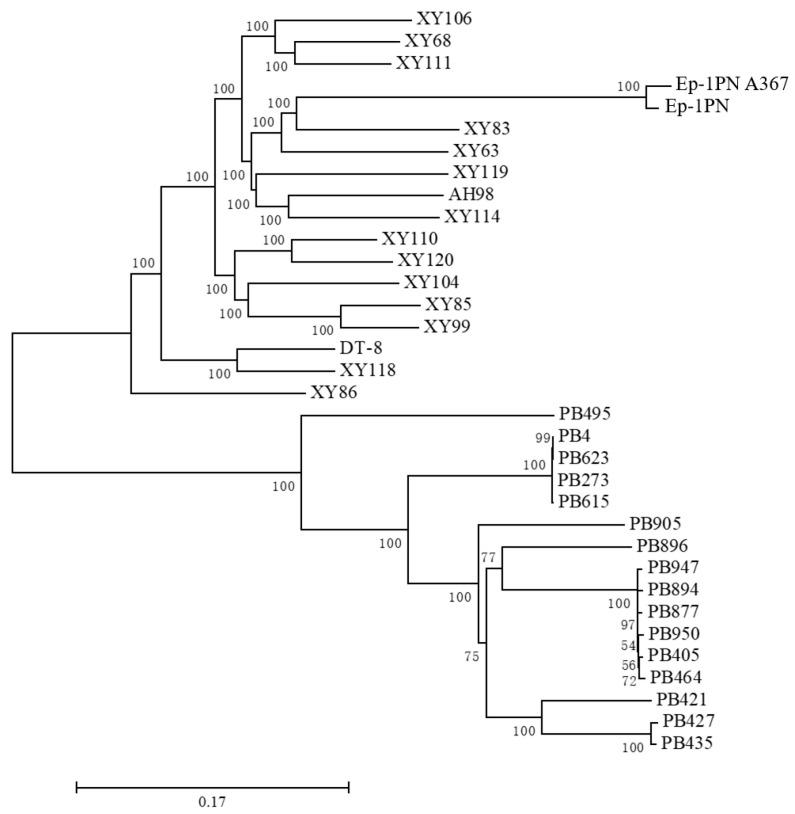
A genome-wide SNP-based phylogenetic analysis for all *S. sclerotiorum* strains in this study. An SNP-based neighbor-joining (NJ) tree was constructed. For details, see Methods. Branch length corresponds to genetic distance based on the SNP number between each other. Bootstrap values (%) obtained with 1000 replicates are indicated on branches. The scale bar corresponds to a genetic distance of 0.1.

To examine the phylogenetic relationship of the genome-sequenced strains tested, a phylogenetic analysis based on all the SNP sites was performed using the MEGA program and a phylogenetic tree based on SNPs was constructed. The strains were divided into two large groups, one containing all Chinese strains and another including all American strains (Figure 4). This result suggested that *S. sclerotiorum* in China and the USA have been separated evolutionarily for a long time. 

There was obvious genomic diversity among Chinese strains. The 14 strains from a single rapeseed field at Xinyang showed diverse SNPs with almost all strains significantly different from each other, and some strains were even grouped with strains from other regions, for example, strain AH98 from Hefei, Anhui province was grouped with a Xinyang strain XY114, strains XY86 and XY83 were grouped with strain DT-8 from Yiyang, Hunan province and Ep-1PN from Jiamusi, Heilongjiang province, respectively (Figure 4).

However, the SNP-based genome diversity of strains from the USA was not rich, it could be roughly divided into three groups. The first group had only one strain, the second included four strains, and the other strains composed the third group. As represented by SNP numbers, the genomes of the four strains in the second group were almost identical to that of strain 1980. Subgroups could be found in the third group: strains PB427 and PB435 might share a set of genome with strain PB421, while strains PB405, PB464, PB877, PB894, PB947, and PB950 shared another set of genome.

### 3.4. The SNP of Offspring Compared with the Parent Strain PB4

**Figure 5:**
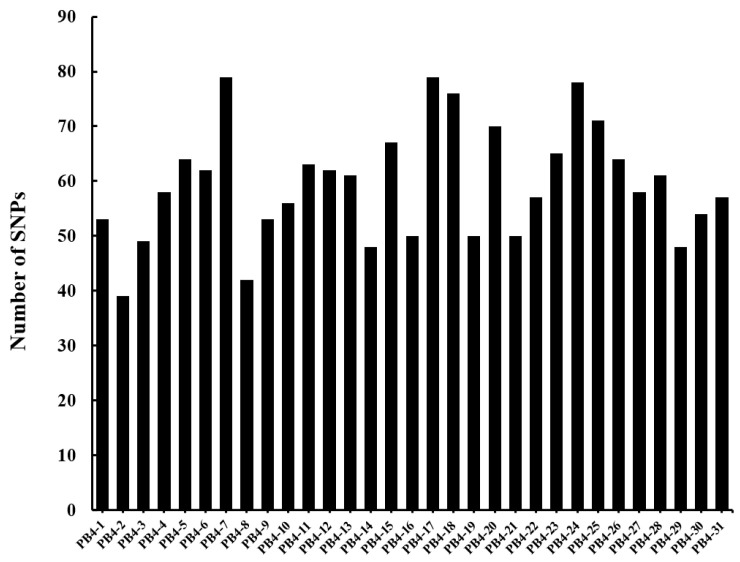
SNPs in the single-ascospore-isolation offspring of strain PB4 compared with strain PB4.

Thirty-one single-ascospore-isolation offspring of strain PB4 were obtained. The growth rate, virulence, and colony morphology of the offspring did not change significantly from the parent strain PB4 (Data not displayed). All 31 offspring were subjected to genome sequencing analysis by using NGS and compared with strain PB4. The results showed that SNPs were found in 435 loci on 21 contigs and the number of SNP in the offspring was 39–79 with 59 on average (Figure 5). Interestingly, 53.1% of the SNPs (231) occurred on Contig_20. The SNPs in the 31 offspring were divided into two groups: occurred on Contig_20 and occurred on other contigs. Fisher’s exact test showed that there was a significant difference between the frequency of SNP on Contig_20 and among the whole genomes (*p* < 0.01).

**Figure 6:**
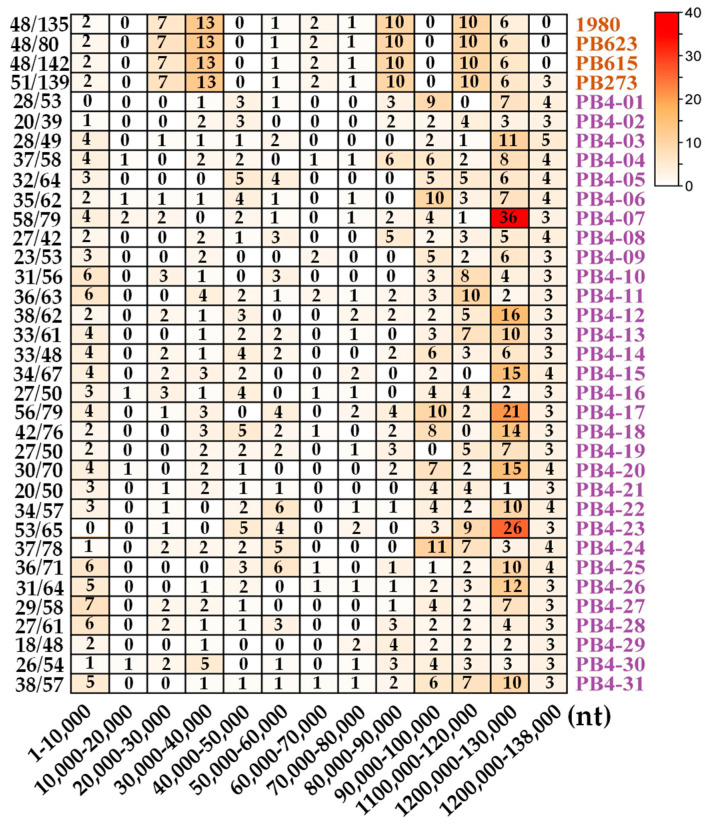
Heatmap of the number of SNP on Contig_20 in strain PB4 compared with the genome of strains listed on the right. The *X*-axis represents the position on Contig_20. The *Y*-axis represents the ratio of SNP on Contig_20/total SNPs. Strains 623, 615, and 273 were isolated in the field and strains PB4-1 to PB4-31 were sexual offspring of strain PB4.

As a hotspot, the mutation position within Contig_20 was counted. The mutation sites in strain PB4 were concentrated on nt 20,000 to 40,000, nt 80,000 to 90,000 and nt 100,000–110,000 compared with strains isolated in the field (Figure 6). Notably, the SNPs caused by sexual reproduction are mainly concentrated at nt 90,000–138,000 and a few single-ascospore-isolation offspring have more mutations at nt 110,000–120,000 (Figure 6).

## 4. Discussion

In this study, we sequenced and assembled a genome of a strain PB4 of *S. sclerotiorum* isolated from a sclerotium mixed in seeds of pinto bean collected by Central Bean Company, Quincy, WA, and unexpectedly found that the genome of strain PB4 is almost identical to that of the reference strain 1980 isolated from diseased bean culls nearly 40 years ago in western Nebraska, USA. We further found that three other strains isolated from the same sclerotium mixture are much closer to strain 1980 even than strain PB4 at the genome level. Comparing the strains from China and the USA, it was found that Chinese strains, even from a single small rapeseed field, have much richer SNPs than the USA strains, suggesting that *S. sclerotiorum* population of China and the USA carried out independent evolution routes. The considerable SNP diversity of strain Ep-1PN and its sexual offspring Ep-1PNA367 which was isolated 25 years ago showed that the genome represented by strain Ep-1PN had an extremely fast variation speed even under lab conditions on PDA medium routinely. Our study here may provide a new vision for the understanding of the genome evolution of *S. sclerotiorum* and even of other organisms that in a specific species, some groups are constantly changing, while others tend to be stable. 

*Sclerotinia sclerotiorum* is rich in genetic diversity worldwide [69,70,71,72,73,74,75,76]. Previously, the Chinese *S. sclerotiorum* population was found to have a greater genetic variance for most of the traits than the US population and they did not share any mycelial compatibility groups or multilocus haplotypes [46]. Our study exhibited directly the rich genetic diversity of Chinese strains isolated from a single rapeseed field by comparing SNPs in the genome. As a homothallic fungus that can carry out an asexual life cycle, clonal strains of *S. sclerotiorum* should not be difficult to obtain in the field [72,77]. In this study, the American strains have less diversity, suggesting that clonality is a major style for *S. sclerotiorum* in the sampled place, which is highly consistent with the previous findings in New York State, USA [47]. The biggest question left over is why Chinese strains have so rich genetic diversity, while the American strains share highly similar genomes. The possible explanation may be associated with the history of farming activities and the cropping system. Agricultural activities enable pathogens to have enough hosts for mass reproduction, while human activities, such as travel, migration, and trading of agricultural products are also the main way of long-distance transmission of pathogens. China has an agricultural history of more than 10,000 years. The hosts of *Sclerotinia*, such as beans and cruciferous crops, are also major traditional food and vegetable crops, which gives *S. sclerotiorum* the opportunity to spread in China year after year.

The fact that strains are very similar to those they were 40 years ago Indicates that the genome of these strains is very stable and the population represented by strain 1980 is widely distributed. The best explanation is that sclerotia of *S. sclerotiorum* spread by mixing in the seed of beans for a long distance. However, this event is unlikely to have occurred in the near recent past since strain 1980 was isolated 40 years ago. Considering that ascospores are the main inocula on beans and other crops, strain PB4 and other strains should have performed sexual reproduction alone at least 40 times in the field. Thus, descendants of strain 1980 should have accumulated more SNPs than those in the genome of strains PB4, PB273, PB615, and PB623. In fact, this may not be the case. Strain 1980 and PB strains have established their own communities at a distance of 2000 km from each other. From the rich SNP diversity of the offspring, it can be seen that strain PB4 may have a similar rate of variation to other strains. Of course, there is also a possibility that the sclerotia of *S. sclerotiorum* can stay awake for a long time and can germinate myceliogenically to infect directly plants. However, unlike ascospores with super quantity and convenience for air transmission [44], sclerotia could not be transmitted widely since its amount formed on plants is limited, e.g., 160 sclerotia in a single diseased cabbage head [78] and be impossible to become a dominant group in such a short time.

Genome stability is crucial to maintain the characteristics of species, while the acquisition of mutations plays a key role in species adaptation [24]. There are mutation hotspots and mutation coldspots in the genome, and these non-random mutation rates indicate that species have an evolutionary risk management strategy to reduce harmful mutations [79]. *Escherichia coli* cultures maintain a stable subpopulation structure during long-term evolution [80]. In this study, we identified a mutation hotspot in the genome of *S. sclerotiorum* strain PB4. When compared with strains 1980, PB273, PB615, and PB623, most SNPs in strain PB4 focused on nt 30,000–40,000, nt 80,000–90,000, and nt 100,000–110,000 within Contig_20. Among sexual offspring of strain PB4, 53.1% of SNPs occurred on Contig_20, which is also significantly distinguished from the overall mutations distributed in other contigs. However, for another pair of strains, Ep-1PN and its sexual progeny Ep-1PNA367, it is hard to identify a mutation hotspot. Even though strains 1980 PB273, PB615, and PB623 might share the same recent ancestor with strain PB4, it is surprising that PB4 could maintain such a stable genome for more than 40 years despite having a mutations hotspot during sexual reproduction.

Sixteen strains isolated from sclerotium mixture in pinto bean seeds might share the same recent ancestor with strain 1980 while with different mutation rates. Obviously, the genome represented by strain 1980 and four PB strains is very stable. However, other sets of genomes phylogenetically distant from that of strain 1980 are also found in the field. Therefore, there might be two types of genomes, one is very stable, like strain 1980 and PB strains; another is unstable and tends to mutate. The forces driving genomic mutation include endogenous DNA polymerase errors, endogenous DNA damage, DNA damage caused by exogenous agents, and the activity of error-prone polymerase, among which oxidative DNA damage and repair significantly affect the mutation rate or spectra [5,81,82]. Recently, a mutator gene was identified in *Saccharomyces cerevisiae* [83]. On the other hand, the key to keeping the genome stable is the repair mechanism, e.g., nucleotide excision repair proteins, which play an important role in clearing oxidative DNA damage [6]. DNA mismatch repair (MMR) deletion leads to a 100-fold increase in mutation frequency [84]. Some studies have shown that organism itself has the function of controlling the mutation rate or has optimized the mutation rate to reduce the harm caused by harmful mutations [79,80]. Because of the negative impact of most mutations on individual adaptability, natural selection is considered to be generally effective in reducing mutation rates (MR). Limited by population size, sexual reproduction will always lead to a decrease in MR, thus maintaining the stability of the genome [85,86,87]. By comparing the genomes of parents and the offspring, it was found that some species have very stable genomes [1,88,89]. Both strains 1980 and PB4 have a mutator gene (mutator Donald Robertson) and a set of MMR genes, considering over 4000 SNPs between strain Ep-1PN and its sexual offspring Ep-1PNA367 which was derived 25 years ago, the genome represented by strains 1980 and PB4 is quite stable. The discovery of identical genomes of strains isolated at a distance of 2000 km away and 40 years apart indicates that there still exist little-known rules in genome stability and variation and prompts us to rethink the traditional understanding of the genetic variation of species. The mechanism underlying this phenomenon may help to develop a technique to stably retain the genetic material of crops, domestic animals, and other useful organisms.

## 5. Conclusions

In this study, a high-quality genome of a new *S. sclerotiorum* strain PB4 was assembled using NGS and SMRT technology. We found that the genome of this strain and the other three strains isolated from the same group of sclerotia was almost identical to the genome of the reference strain 1980, though they were isolated apart for almost 40 years and at a distance of 2000 km from each other. This phenomenon is rather rare since it exhibited a scenario where extremely stable genomes and rapidly mutated genomes coexist in the same species. This indicates that there may be an unknown mechanism in the field to stabilize some genomes of species. Here we report the existence of genomic stability in the USA; whether it is universal in *S. sclerotiorum* population in other regions or in other life species is to be further investigated.

## Figures and Tables

**Table 1:** List of *Sclerotinia sclerotiorum* strains used in the study.

Strains	Collection Date	Host	Geographical Origin	Reference
1980	1980	Bean	Western, Nebraska, USA	[49]
PB4	Oct 2018	Pinto bean	Quincy, Washington, USA	This study
PB273	Oct 2018	Pinto bean	Quincy, Washington, USA	This study
PB405	Oct 2018	Pinto bean	Quincy, Washington, USA	This study
PB421	Oct 2018	Pinto bean	Quincy, Washington, USA	This study
PB427	Oct 2018	Pinto bean	Quincy, Washington, USA	This study
PB435	Oct 2018	Pinto bean	Quincy, Washington, USA	This study
PB464	Oct 2018	Pinto bean	Quincy, Washington, USA	This study
PB495	Oct 2018	Pinto bean	Quincy, Washington, USA	This study
PB615	Oct 2018	Pinto bean	Quincy, Washington, USA	This study
PB623	Oct 2018	Pinto bean	Quincy, Washington, USA	This study
PB877	Oct 2018	Pinto bean	Quincy, Washington, USA	This study
PB894	Oct 2018	Pinto bean	Quincy, Washington, USA	This study
PB896	Oct 2018	Pinto bean	Quincy, Washington, USA	This study
PB905	Oct 2018	Pinto bean	Quincy, Washington, USA	This study
PB947	Oct 2018	Pinto bean	Quincy, Washington, USA	This study
PB950	Oct 2018	Pinto bean	Quincy, Washington, USA	This study
AH98	2009	Rapeseed	Hefei, Anhui Province, China	[54]
DT-8	2007	Rapeseed	Yiyang, Hunan Province, China	[53]
Ep-1PN	1992	Eggplant	Jiamusi, Heilongjiang Province, China	[52]
Ep-1PNA367	1997	Eggplant	Jiamusi, Heilongjiang Province, China	[52]
XY63	Oct 2020	Rapeseed	Xinyang, Henan Province, China	This study
XY68	Oct 2020	Rapeseed	Xinyang, Henan Province, China	This study
XY83	Oct 2020	Rapeseed	Xinyang, Henan Province, China	This study
XY85	Oct 2020	Rapeseed	Xinyang, Henan Province, China	This study
XY86	Oct 2020	Rapeseed	Xinyang, Henan Province, China	This study
XY99	Oct 2020	Rapeseed	Xinyang, Henan Province, China	This study
XY104	Oct 2020	Rapeseed	Xinyang, Henan Province, China	This study
XY106	Oct 2020	Rapeseed	Xinyang, Henan Province, China	This study
XY110	Oct 2020	Rapeseed	Xinyang, Henan Province, China	This study
XY111	Oct 2020	Rapeseed	Xinyang, Henan Province, China	This study
XY114	Oct 2020	Rapeseed	Xinyang, Henan Province, China	This study
XY118	Oct 2020	Rapeseed	Xinyang, Henan Province, China	This study
XY119	Oct 2020	Rapeseed	Xinyang, Henan Province, China	This study
XY120	Oct 2020	Rapeseed	Xinyang, Henan Province, China	This study

**Table 2:** The count and length of structural variations (SVs) between *S. sclerotiorum* strains PB4 and 1980.

		Size Range 50–500 (bp)	Size Range 500–10,000 (bp)	Total
Insertion	Count	5	6	11
	Length	1150 (bp)	22,676 (bp)	23,826 (bp)
Deletion	Count	2	5	7
	Length	263 (bp)	15,331 (bp)	15,594 (bp)

## Data Availability

All datasets were deposited in NCBI with the genome accession number JANHKL000000000.

## Supplementary Materials

The following supporting information can be downloaded at: https://www.mdpi.com/article/10.3390/jof8111212/s1, Figure S1: The number of CAZymes and PHI-related proteins identified in the genome of *S. sclerotiorum* strain PB4. (A) The number of CAZymes including Glycoside Hydrolases (GH), Glycosyl Trans-ferases (GT), Polysaccharide Lyases (PL), Carbohydrate Esterases (CE), Auxiliary Activities (AA), and Carbohydrate-Binding Modules (CBM). (B) The number of PHI-related proteins. (C) The number of various GO functional categories; Figure S2: The seven new prediction proteins in the genome of strain PB4; Figure S3A: SNPs distributed in each contig of the strain PB4 compared to the strain 1980; Figure S3B: SNPs distributed in each chromosome of the strain 1980 compared to the strain PB4; Table S1: Data statistics of PacBio reads of PB4; Table S2: Data statistics of Illumina reads of PB4; Table S3: The parameters for *de novo* genome assembly and annotation of PB4; Table S4: The detailed information of 135 SNPs between the strains PB4 and 1980, validated by PCR.
