# Supplementary material for: Fungal Strains with Identical Genomes Were Found at a Distance of 2000 Kilometers after 40 Years
Source: J Fungi (Basel). 2022 Nov 16;8(11):1212. doi: 10.3390/jof8111212 (PMC9697809; doi:10.3390/jof8111212)
Supplement: Supplementary file 1 [file jof-08-01212-s001.zip › Table S1.pdf]

Supplementary Data

Table S1. Data statistics of PacBio Reads of PB4

**Features**	***S. sclerotiorum* PB4**
Vaild ZWM number	142,401
Subreads number	12,229,730
Subreads total bases (bp)	12,571,219,210
Subreads mean length (bp)	10,222
Subreads N50 (bp)	10,915
Subreads N90 (bp)	7,150
Subreads max length (bp)	132,037
Subreads min length (bp)	2000
