# Supplementary material for: Fungal Strains with Identical Genomes Were Found at a Distance of 2000 Kilometers after 40 Years
Source: J Fungi (Basel). 2022 Nov 16;8(11):1212. doi: 10.3390/jof8111212 (PMC9697809; doi:10.3390/jof8111212)
Supplement: Supplementary file 1 [file jof-08-01212-s001.zip › Table S2.pdf]

Supplementary Data

Table S2. Data statistics of Illumina Reads of PB4

**Features**	***S. sclerotiorum* PB4**
Insert size (bp)	300-400bp
Reads length (bp)	(150:150)
Raw data (bp)	3,768,320,425
Adapter (%)	0.67
Duplication (%)	0.1
Total reads	24,539,788
Filtered reads (%)	0.8
Low quality filtered reads (%)	0
Clean data (bp)	3,738,624,179
