# Supplementary material for: Fungal Strains with Identical Genomes Were Found at a Distance of 2000 Kilometers after 40 Years
Source: J Fungi (Basel). 2022 Nov 16;8(11):1212. doi: 10.3390/jof8111212 (PMC9697809; doi:10.3390/jof8111212)
Supplement: Supplementary file 1 [file jof-08-01212-s001.zip › Table S3.pdf]

## Supplementary Data

**Table S3. The parameters for *de novo* genome assembly and annotation of PB4**

| **Features** | ***S. sclerotiorum* PB4** |
|---|---|
| Total contig number | 27 |
| Total length (bp) | 39,218,820 |
| Total ungapped length (bp) | 39,218,820 |
| Coverage | 148× |
| Contig N50 length (bp) | 2,127,701 |
| G+C content (%) | 41.52% |
| Repeat (%) | 11.47% |
| Number of gene | 11,248 |
| Number of CDS | 32,363 |
| Number of exon | 32,358 |
