# Supplementary material for: Fungal Strains with Identical Genomes Were Found at a Distance of 2000 Kilometers after 40 Years
Source: J Fungi (Basel). 2022 Nov 16;8(11):1212. doi: 10.3390/jof8111212 (PMC9697809; doi:10.3390/jof8111212)
Supplement: Supplementary file 1 [file jof-08-01212-s001.zip › Table S4.pdf]

Supplementary Data

Table S4. The detailed information of 135 SNPs between the strains PB4 and 1980, validated by PCR

Contig	Loc	Ref	Alt
Contig_01	648914	C	G
Contig_01	1211833	A	T
Contig_01	1223202	A	T
Contig_01	1360126	C	A
Contig_01	1386765	A	G
Contig_01	1480975	G	A
Contig_01	1807474	A	G
Contig_01	2467981	G	T
Contig_02	55829	T	C
Contig_02	120910	A	G
Contig_02	141276	T	C
Contig_02	141299	C	A
Contig_02	143303	C	T
Contig_02	1075603	G	A
Contig_02	2690190	T	C
Contig_02	3538562	C	T
Contig_03	492143	T	C
Contig_03	2004938	C	T
Contig_04	311272	C	T
Contig_04	316637	G	A
Contig_04	760240	A	T
Contig_04	1276533	C	T
Contig_04	1386143	A	G
Contig_04	1574153	G	C
Contig_05	535099	C	A
Contig_05	1586382	A	G
Contig_05	1592443	T	C
Contig_05	1597463	C	T
Contig_05	1620463	A	G
Contig_05	1950571	T	C
Contig_05	2124936	C	T
Contig_06	617452	T	C
Contig_06	667981	C	T
Contig_06	953645	T	C
Contig_06	1127348	A	G
Contig_06	2081689	G	C
Contig_07	393753	G	A
Contig_07	583298	C	T
Contig_07	1561543	T	C
Contig_07	2003474	T	C

Contig	Loc	Ref	Alt
Contig_08	332251	A	C
Contig_08	1713551	G	A
Contig_08	2002372	G	A
Contig_08	2035397	A	T
Contig_09	103515	T	C
Contig_09	145080	C	T
Contig_09	423108	G	A
Contig_09	1202424	A	G
Contig_09	1505290	T	C
Contig_10	638306	A	G
Contig_10	697517	C	T
Contig_10	983966	G	A
Contig_10	1147759	A	T
Contig_10	1487138	C	A
Contig_11	95043	A	G
Contig_11	133068	C	T
Contig_11	343970	G	T
Contig_11	346813	G	A
Contig_11	523676	G	A
Contig_11	693530	A	T
Contig_11	839911	G	A
Contig_11	1225727	A	T
Contig_12	355901	T	A
Contig_12	1468883	A	G
Contig_13	1096797	A	C
Contig_13	1116797	G	A
Contig_13	1570953	C	T
Contig_13	1640338	A	G
Contig_13	1707515	T	C
Contig_14	328295	G	A
Contig_14	408901	T	C
Contig_14	704840	T	A
Contig_14	916221	A	C
Contig_15	79199	G	A
Contig_15	163825	C	T
Contig_15	211885	C	T
Contig_15	289608	C	T
Contig_15	803437	T	A
Contig_16	579926	T	C
Contig_16	817690	A	G
Contig_18	5953	T	C
Contig_18	17826	T	C
Contig_18	206637	G	T

Contig	Loc	Ref	Alt
Contig_19	65856	T	C
Contig_19	184259	C	T
Contig_20	4056	C	T
Contig_20	28487	G	C
Contig_20	28630	C	T
Contig_20	28685	T	C
Contig_20	29169	A	G
Contig_20	29864	G	A
Contig_20	29920	G	C
Contig_20	30084	A	G
Contig_20	30439	G	T
Contig_20	31412	G	A
Contig_20	31971	CT	C
Contig_20	32075	G	A
Contig_20	32081	C	A
Contig_20	32651	C	T
Contig_20	32985	C	T
Contig_20	35775	G	A
Contig_20	36469	T	C
Contig_20	37644	C	T
Contig_20	37754	A	G
Contig_20	38738	T	C
Contig_20	38888	C	T
Contig_20	58871	A	G
Contig_20	60603	T	A
Contig_20	60783	C	T
Contig_20	77854	G	A
Contig_20	80564	A	T
Contig_20	80786	A	G
Contig_20	82022	T	C
Contig_20	85000	G	A
Contig_20	85005	T	C
Contig_20	85220	C	T
Contig_20	85238	C	T
Contig_20	85265	C	T
Contig_20	87207	C	G
Contig_20	88882	A	T
Contig_20	103329	T	G
Contig_20	103408	T	G
Contig_20	103883	C	T
Contig_20	104016	G	A
Contig_20	104613	T	G
Contig_20	104657	C	G

Contig	Loc	Ref	Alt
Contig_20	105543	C	A
Contig_20	105632	T	C
Contig_20	105760	C	T
Contig_20	105975	T	C
Contig_20	110552	T	C
Contig_20	110589	G	A
Contig_20	110639	A	C
Contig_20	110710	T	C
Contig_22	40391	G	A
