# Supplementary figures and images for: Fungal Strains with Identical Genomes Were Found at a Distance of 2000 Kilometers after 40 Years
Source: J Fungi (Basel). 2022 Nov 16;8(11):1212. doi: 10.3390/jof8111212 (PMC9697809; doi:10.3390/jof8111212)

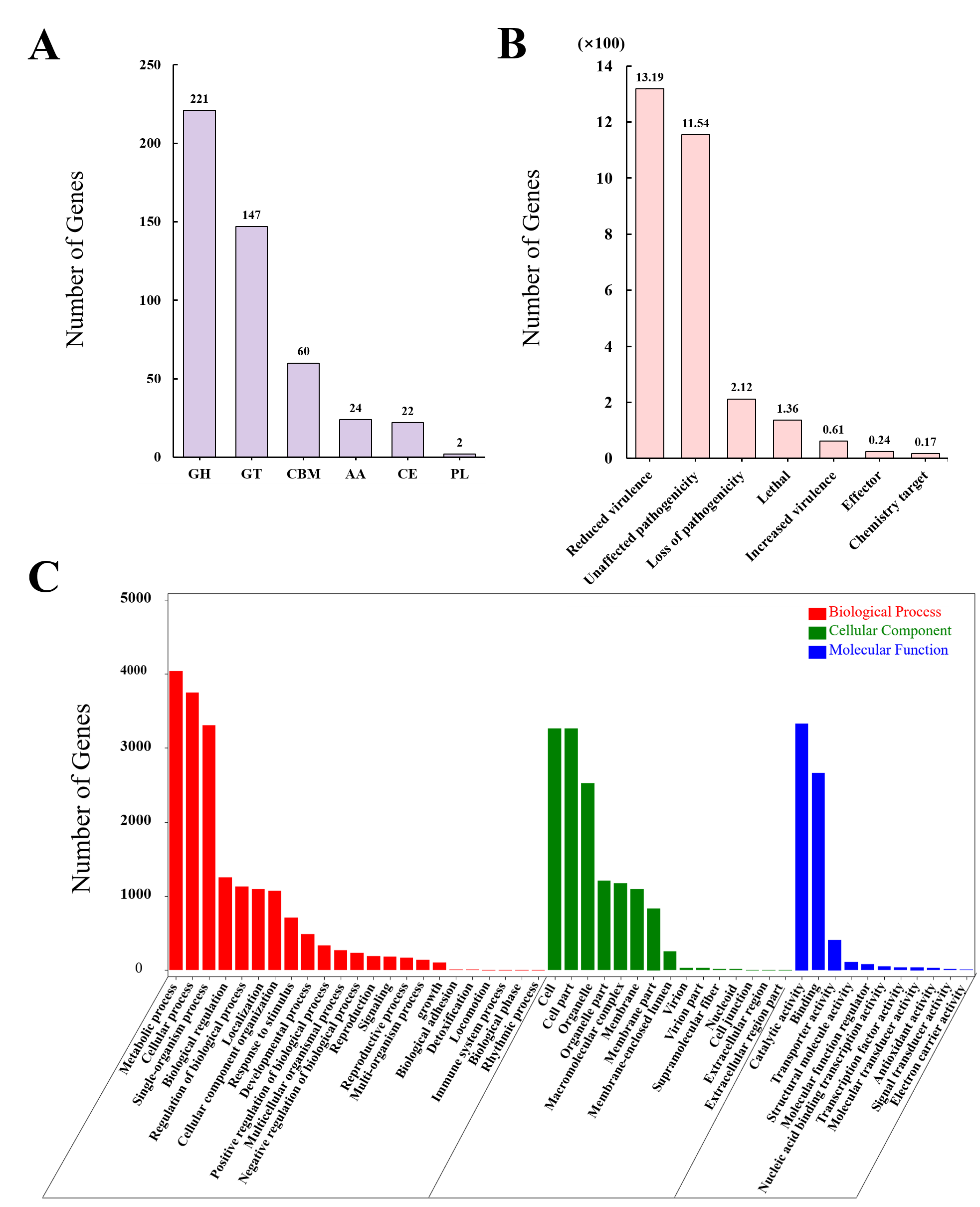

Supplement: Supplementary file 1 [file jof-08-01212-s001.zip › Figuer S1.png]

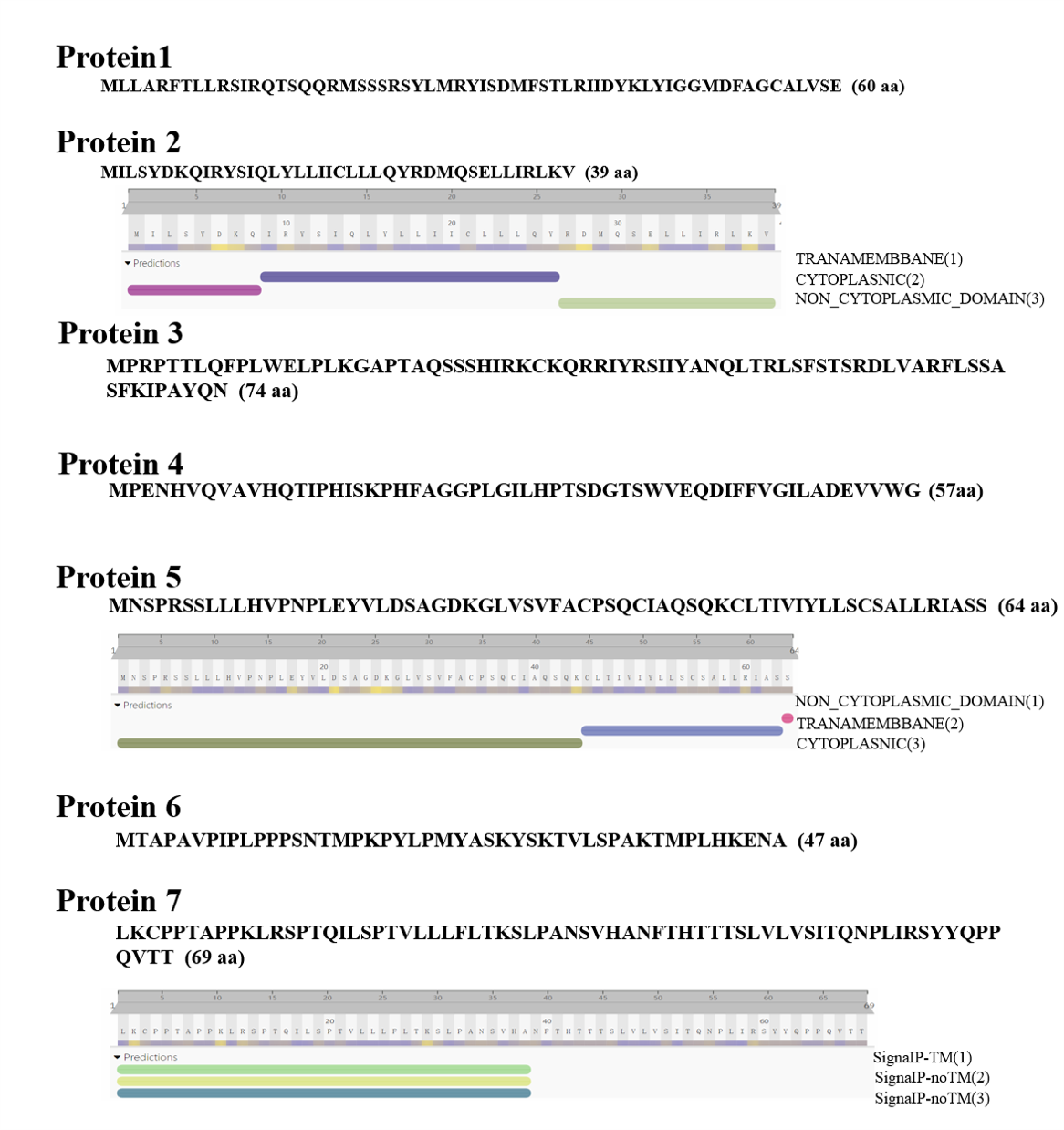

Supplement: Supplementary file 1 [file jof-08-01212-s001.zip › Figuer S2.png]

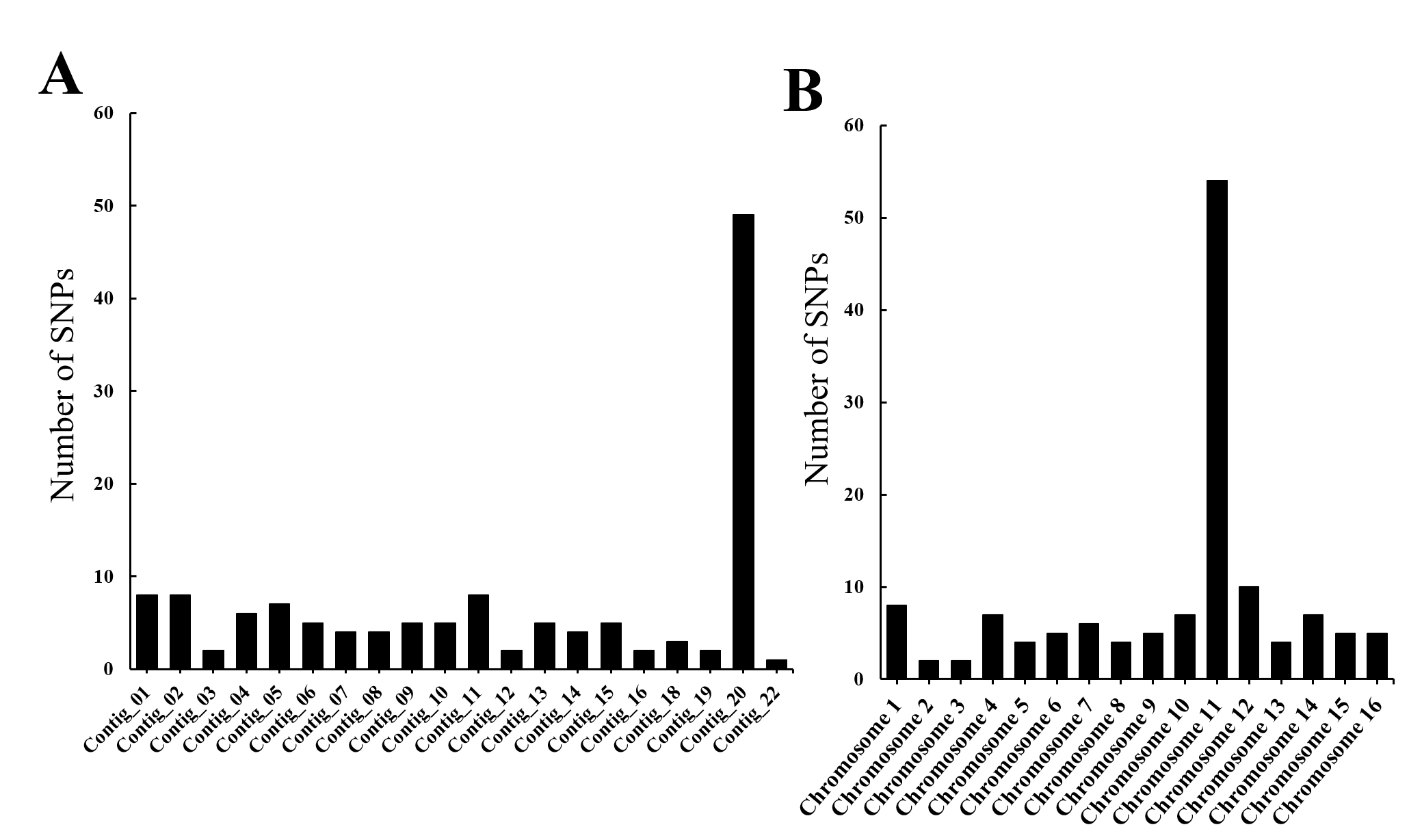

Supplement: Supplementary file 1 [file jof-08-01212-s001.zip › Figuer S3.png]
